# Advancing Trauma Care in Sri Lanka: System Overview and Developmental Priorities

**DOI:** 10.7759/cureus.82674

**Published:** 2025-04-21

**Authors:** Kayaththery Varathan, Havil Stephen Alexander Bakka, Tharaga Kirupakaran

**Affiliations:** 1 Orthopaedics and Trauma, Barts Health Trust, London, GBR; 2 Neurosurgery, Royal Sussex County Hospital, Brighton, GBR; 3 Acute Medicine, Kingston Hospital NHS Foundation Trust, Kingston, GBR

**Keywords:** disaster medicine, emergency medical services (ems), prehospital care, public health policy, road traffic accidents (rtas), sri lanka healthcare system, trauma care in low-income countries (lics), trauma registry, trauma rehabilitation, trauma systems

## Abstract

Trauma is a leading cause of global mortality and morbidity, with road traffic injuries being a significant contributor, especially in low- and middle-income countries (LMICs). With 25,000 road traffic accidents annually, Sri Lanka faces a substantial trauma burden, making it the leading cause of hospitalisation. Effective trauma systems, encompassing prevention, pre-hospital care, in-hospital treatment, rehabilitation, and planning, are crucial for improving patient outcomes and alleviating strain on the healthcare system. This review examines the key components of trauma systems and evaluates Sri Lanka’s current trauma care infrastructure, identifying gaps and areas for improvement. Despite initiatives such as injury prevention programs, the establishment of pre-hospital ambulance services, and the introduction of emergency medicine specialists, Sri Lanka lacks a well-defined trauma pathway, standardized protocols, and adequate training for emergency medical technicians (EMTs). The recent development of a trauma registry in the northern region highlights the need for improved data collection and resource allocation. Challenges such as poor communication, limited public awareness of emergency services, and insufficient EMT training hinder the effectiveness of pre-hospital care. To strengthen its trauma system, Sri Lanka must prioritize the implementation of national trauma policies, enhance training programs, improve communication pathways, and expand the trauma registry nationwide. Addressing these issues requires recognising the multifactorial nature of implementing systemic improvements, which includes financial investment, political commitment, and a coordinated effort to create a standardized, efficient trauma care system that can reduce mortality and improve outcomes for trauma patients.

## Introduction and background

Trauma remains a significant contributor to global mortality and morbidity, leading to an annual death toll of 4.4 million [1]. This surpasses the combined death toll from tuberculosis, malaria, and HIV [1]. A leading cause of deaths, especially in low- and middle-income countries (LMICs), is road traffic injuries, resulting in up to 90% of all trauma admissions [2].

Sri Lanka records 25,000 road traffic accidents annually, leading to 10 deaths per day. With 3,100 trauma-related hospital admissions per 100,000, it remains the leading cause of hospitalisation in Sri Lanka [3]. These statistics highlight the immense burden trauma places on healthcare. In such circumstances, trauma systems are essential for enhancing patient outcomes and easing the strain on hospitals. However, the effectiveness of these systems varies across regions. This review will examine the key components of an effective trauma system, which include prevention, pre-hospital care, in-hospital treatment, rehabilitation, and planning. It will also explore potential improvements for Sri Lanka.

## Review

Elements of a trauma system

A trauma system is defined as a synchronised, planned pathway in a geographic area that provides a full range of care to injured patients and is integrated within the health system [4]. Most trauma systems include prevention, pre- and post-hospital care, and acute care in local or major trauma centres [5]. However, there are differences in these systems that arise due to the varying economic wealth of countries and the resources they have available. Trauma care in Sri Lanka originated in 1965 with the establishment of the trauma centre at the Colombo General Hospital (CGH) [6]. CGH is the only hospital in the country that meets the trauma centre standard of high-income nations in regard to resources. CGH managed at least 50 mass casualty incidents (MCIs) from 1991 to 2009. However, despite its experience in handling MCIs over the years, it lacks a well-defined trauma pathway and struggles particularly with effective triage and transfer to regional hospitals. This subsequently leads to ineffective use of hospital beds and results in overcrowding [7].

Prevention

Prevention programmes have been shown to reduce injuries, thereby lowering morbidity and mortality. This leads to lower healthcare costs, which can be particularly important in LMICs. Sri Lanka released national guidelines on injury prevention in 2010 in response to the increasing injury burden [8]. Simple measures such as retroreflectors on bicycles and reflective clothing have also been emphasised since then.

Lakmal et al. presented data from the second-largest trauma centre in the capital in 2021 with 473 casualties, which revealed that the most common causes of road traffic accidents (RTAs) included alcohol consumption, illegal driving without a licence, and motorcycle riders and passengers not wearing helmets [9]. Studies prior to this, dating as far back as 1997, have also shown similar incidences and causes of these injuries [10,11]. This raises the question of why simple measures such as stricter law enforcement have seen severe non-compliance and poor governance.

Beyond RTAs, another preventable injury burden in Sri Lanka comes from burn injuries, with 10,000 victims per year, largely due to the use of unsafe lighting sources in low-income areas. The financial strain is significant, with Sri Lanka spending $10,000 every four days on burn treatments. These are funds that could be redirected to preventive measures [8]. This is due to the use of kerosene lamps, particularly in shanty towns with no electricity [12]. This has been addressed by the "Safe Bottle Lamp Foundation", introduced in 1992, which has created safe lamps and received approval from the World Health Organization. To date, 21% of these bottles have been replaced, leading to a reduced number of burn presentations, along with increased electricity provision across Sri Lanka [13].

Prehospital care

Prehospital care, defined as the period from the point of injury to the initiation of hospital management, is a vital component of the trauma system [8,14]. Patients who are transferred to the hospital rapidly, allowing for treatment to occur within the golden hour, tend to have better outcomes than those with delayed admissions. The golden hour is crucial, as patients following trauma must reach a centre within 60 minutes. This allows for the commencement of prompt resuscitation care, improving patient outcomes [15]. This is supported by Hsieh et al., who studied 963 trauma patients and concluded that treatment within the first two hours is associated with better patient survival rates [16]. Therefore, those who provide this service must also be equipped with adequate skillsets. These services are also essential during transfers between trauma centres.

Prehospital services in Sri Lanka consist of a range of programmes including hospital-based services, local government-operated services, NGO services, and private services [17]. The expansion of prehospital services in Sri Lanka was catalysed by the 2004 tsunami, the country's deadliest natural disaster, leading to the death of 31,000 and a further 1.3 million being affected [18].

The prehospital sub-committee works to combine these services into a single harmonised national approach [19]. The first prehospital ambulance service was initiated in 2007 with the introduction of the free call number 110, which was initially available only in the capital, Colombo, due to financial and infrastructure constraints. This later expanded in 2016 when the 1990 Suwa Seriya free ambulance service was established, with 687 ambulances operating across the island and an average response time of 8 to 12 minutes. This service has allowed for patients to be assessed en route, enabling a smooth handover to physicians, and the ambulances are able to carry out certain investigations such as ECGs when indicated [20,21].

Unlike in many high-income countries, where EMTs undergo extensive paramedic training, Sri Lankan first responders are primarily trained in basic rescue techniques, CPR, oxygen administration, and splinting [22].

A study looking into the modes of transport to treatment centres in Northern Sri Lanka in 2020 highlighted that three-wheelers were the leading mode of transport. This reliance on three-wheelers, rather than ambulances, raises concerns about delayed definitive care, lack of en route medical interventions, and potential worsening of injuries during transport. Only 25% received any first aid prior to arrival at hospital [3].

Acute care

Emergency departments are the first point of care for trauma patients and provide care 24/7. The presence of emergency medicine consultants onsite is valuable to provide senior-led input. This leads to improved trauma care by enhancing injury assessment and reducing clinical errors [23].

Despite the benefits of consultant-led emergency care, Sri Lanka historically lacked dedicated emergency medicine specialists, relying instead on a multi-specialty approach. Until 2019, Sri Lanka had no formally trained emergency medicine doctors. The emergency department was instead run by a team of various specialties including general physicians, orthopaedic surgeons and anaesthetists [22]. The emergency department is still run with the aid of other specialties due to only 30 emergency physicians across the country in 2022. In 2016, the Ministry of Health created guidelines for emergency care services [24]. While these guidelines clarify the need for initial observations/investigations, a triage system, and establish a four-hour waiting time target, they do not mandate consultant presence in emergency departments or provide standardised resuscitation protocols.

Protocols/guidelines

Guidelines are a significant part of a trauma system as they act as a referral guide for all those involved. In well-established systems such as London, protocols cover before, during, and after hospital stay [25]. Additionally, they allow for care to be uniform, providing the best patient service regardless of how major the trauma.

In the absence of a national framework, despite the country having experienced natural disasters, a civil war lasting 26 years, and a recent Easter terrorist bombing [26], hospitals in Sri Lanka have adopted a combination of injury-specific protocols and international guidelines such as NICE. Similar challenges have been observed in other LMICs, where lack of funding and political will have delayed the implementation of standardised trauma systems. Many hospitals rely on injury-specific guidelines, such as those for chest and abdominal trauma, and adopt international protocols like NICE due to the absence of national trauma policies. Recognising this gap, the Trauma Secretariat was established in January 2007 to oversee the development of a trauma system [6,8]. Although trauma and emergency care policies were drafted, their implementation has been hindered by financial constraints, inconsistent government prioritisation, and limited infrastructure support.

Rehabilitation

Rehabilitation is a crucial component of trauma aftercare, ensuring that patients receive appropriate assessment and management of their needs [27]. Sri Lanka’s civil war led to advancements in prosthetic and physical rehabilitation, as well as the expansion of counselling and therapy services for war survivors [28]. At the end of the civil war in 2009, a rehabilitation programme for spinal cord injury was implemented. Eighty-nine patients were admitted and assessed before and after with a standardised Spinal Cord Independence Measure II (SCIM). The score had improved from 55 to 71, and 79% achieved clinically improved outcomes [29].

While the civil war primarily drove advancements in physical rehabilitation, the 2004 tsunami played a key role in shaping mental health and trauma counselling programmes. The 2004 tsunami marked a turning point for mental health awareness in Sri Lanka, leading to the establishment of formal trauma counselling services and long-term psychological support initiatives [30]. Both events significantly influenced the expansion and improvement of physical therapy services. Although these events spurred improvements, access to rehabilitation services remains uneven across different regions of Sri Lanka.

Areas for improvement

Trauma Registry Development

In 2020, Sri Lanka witnessed an increase of 85% in RTAs [31]. To combat this crisis, the Northern region of Sri Lanka developed a local electronic registry to understand the determinants and occurrence of injury [3]. This is particularly useful in LMICs, as it can help identify where to apply the limited funding tactfully to tackle the trauma crisis and lower the number of casualties.

A three-month data collection period helped understand the demographics, vehicles involved in the injury, and the mode of transport to the hospital. The pilot study highlighted that only 52% of patients arrived within two hours, with the main mode of transport being three-wheeled vehicles [3]. A quarter of patients took longer than two hours to arrive at the hospital. Limb injuries were the most common type, which subsequently meant fracture stabilisation was the most recurrent management required [3].

Findings from the registry align with global research on trauma care disparities. For instance, a study by Sharma et al. demonstrated that patients in high-income countries (HICs) are six times more likely to survive life-threatening but potentially survivable injuries due to faster hospital access [32].

The registry has not only identified critical gaps in trauma care but has also informed strategic improvements, such as optimising ambulance distribution, integrating GPS tracking, and advocating for paramedic training [3]. However, full implementation remains a challenge due to financial and logistical barriers. These include organising ambulances in close proximity to accident-prone areas, GPS in ambulances, and statistics for the government to understand the importance of paramedic training and possibly first aid training for three-wheeled drivers [3].

Expanding the trauma registry nationwide will require overcoming logistical and financial barriers, including software procurement, hardware upgrades, and reliable internet access in rural hospitals. Additionally, increasing the number of trained data collectors and ensuring continuous monitoring will be essential for long-term sustainability. This includes providing software and hardware availability and upgrades where indicated across the country. More data collectors must be employed and trained for optimal data extraction. Frequent monitoring will be required to assess whether the interventions introduced are lowering the number of casualties [33].

Communication

Only 25% of the Sri Lankan residents are aware of the emergency number 110 [34]. It implies minimal effort in creating awareness of the above, and many still rely on private transport. Medical dispatchers only undergo a three-hour introductory course, in comparison to a five-week classroom training course and 10 supervised shifts in the UK [17,35].

As a result, many technical errors have emerged, leading to a lack of professional communication. This leads to errors in dispatch and inefficient use of resources. Furthermore, emergency first responders often fail to communicate details about the patient they are transporting to the hospital. Even when communication is successful, the information may not reach the appropriate staff. As a result, patients are sometimes transferred to alternative nearby centres instead of the most appropriate facility.

Breakdowns in communication delay critical interventions, misallocate resources, and can ultimately worsen patient outcomes. Ensuring a streamlined and structured communication pathway is essential for optimising trauma care. More awareness, training, and modifications need to be made to create a centralised communication centre. Residents should be aware of the emergency number and free government-funded ambulances. A centralised dispatch system, incorporating both public and private ambulances, could ensure better patient tracking and reduce delays. Financial incentives or policy mandates may be necessary to encourage private providers to participate.

Emergency dispatchers require more than three hours of training and should have a pathway to work from. Emergency technicians must alert the hospital in advance with a brief history. This allows the hospital to be prepared for the incoming emergency. Improving this will hopefully reduce the transit time of the patient, allowing them to be seen within the “golden hour”.

Training

Currently, EMTs are only trained to levels 1 and 2. Level 1 includes basic and advanced first aid skills such as cardiopulmonary resuscitation and splinting, while level 2 includes teaching about oral/nasal airway adjuncts and defibrillators. They are not trained to levels 3 and 4, which is equivalent to the standards of a paramedic in the United States. Therefore, the EMTs are not equipped to administer intravenous therapy, paediatric life support, cardiac life support, and other procedures such as chest tube insertion or intubation. Although an additional 2,500 were employed and trained as EMTs post-tsunami in 2004, there has been no sustained effort since. Thus, in many regions of Sri Lanka, ambulances function as a mode of transport, where workers have no insight into life-threatening injuries or initial management to stabilise patients. This makes the ambulances no different from three-wheelers. A standardised system needs to be created and put in place to recruit EMTs and recognise the importance of paramedics [22].

There is no literature available on the impact of introducing emergency medicine specialty training programmes in Sri Lanka. Although emergency medicine specialty training is well established in Western countries, its impact in Sri Lanka remains undocumented. Research is needed to determine whether its implementation has improved patient care efficiency or introduced delays due to increased referrals to other specialties. The National Hospital of Sri Lanka in Colombo serves as the country's largest trauma centre, treating 50,000 patients annually [36]. There are no policies or guidelines as to how patients are transferred here and how trauma calls are conducted, if any. In view of the 2004 tsunami, a Disaster Management Act was created in 2005, which highlights the collaboration between various authorities. However, the Act primarily focuses on humanitarian aid, such as shelter, food, and clothing, with limited provisions for prehospital emergency medical care and trauma response protocols. Future policy revisions should incorporate structured disaster medical response frameworks.

## Conclusions

Sri Lanka’s trauma system is still in its early stages of development. Over the years, key preventative measures and the importance of prehospital care have been recognised, leading to initial steps for improvement. The recent introduction of the trauma registry is extremely promising, but more efforts will be needed to conduct it annually on a national basis. Enhancing communication, training, and public awareness is essential for improving patient care and strengthening prehospital services. Despite the high burden of trauma cases, Sri Lanka still lacks a standardised trauma care pathway. Implementing a structured system is crucial to ensure consistent, high-quality care nationwide. While financial constraints and political support influence trauma system development, significant progress can still be achieved through better organisation and integration of existing resources. Regular monitoring of system improvements will help pinpoint areas of prehospital care that require greater attention and funding. Establishing a strong foundation for trauma care will not only enhance acute management but also provide insights for strengthening rehabilitation services in the long term.
